# Determination of zones at risk for fasciolosis in the department of Haute-Vienne, central France: a retrospective study on natural infections detected in 108,481 *Galba truncatula* for 37 years

**DOI:** 10.1051/parasite/2017055

**Published:** 2017-12-22

**Authors:** Philippe Vignoles, Daniel Rondelaud, Gilles Dreyfuss

**Affiliations:** INSERM 1094, Faculties of Medicine and Pharmacy, 2 rue du Docteur Raymond Marcland, 87025 Limoges Cedex France

**Keywords:** Altitude, at risk zones, climate, *Fasciola hepatica*, *Galba truncatula*, Haute-Vienne, snails

## Abstract

A retrospective study on the natural infection of *Galba truncatula* by *Fasciola hepatica* was carried out in the French department of Haute-Vienne to determine whether there are areas at risk for fasciolosis. Adult snails included in this analysis came from samples collected from pastures on 259 farms and from 121 wild watercress beds between 1970 and 2006. *Fasciola hepatica* infection rates were examined in relation to altitude and climatic data (mean annual rainfall, mean annual temperature) of each municipality. In a total of 108,481 snails collected in 151 municipalities, the overall prevalence of infection was 3.8% but varied according to the municipalities from which samples were taken (from 1% to 7.4%). The prevalence of *F. hepatica* infection in snails significantly decreased when the mean altitude of municipalities or their mean annual rainfall increased. However, this prevalence significantly increased with increasing mean annual temperatures. Studying the prevalence of infection in these snails makes it possible to delineate zones at risk for fasciolosis on the acid soils of Haute-Vienne. The risk of infection for livestock would be greater in areas of Haute-Vienne below 400 m above sea level and would gradually decrease when the altitude of the land increases.

## Abstract (fr)

Une étude rétrospective sur l'infestation naturelle de *Galba truncatula* par *Fasciola hepatica* a été effectuée dans le département de la Haute-Vienne pour déterminer s'il existe des zones à risque pour la fasciolose. Les limnées adultes impliquées dans cette analyse proviennent d'échantillons prélevés dans les pâturages de 259 fermes et dans 121 cressonnières sauvages entre 1970 et 2006. Les taux d'infection par *Fasciola hepatica* ont été mis en relation avec l'altitude et les données climatiques (précipitations annuelles moyennes, température annuelle moyenne) de chaque municipalité. Sur un total de 108.481 limnées récoltées dans 151 municipalités, la prévalence globale de l'infestation était de 3,8 %, mais variait selon les municipalités dans lesquelles les échantillons ont été prélevés (de 1 % à 7,4 %). La prévalence de l'infestation par *F. hepatica* diminue considérablement lorsque l'altitude moyenne des municipalités ou leurs précipitations annuelles moyennes augmente. D'autre part, cette prévalence augmente considérablement avec l'augmentation de la température annuelle moyenne des municipalités. L'étude de la prévalence de l'infestation chez ces limnées permet de délimiter des zones à risque pour la fasciolose sur les sols acides de la Haute-Vienne. Le risque d'infestation pour le bétail serait plus élevé dans les zones de la Haute-Vienne situées en dessous de 400 m d'altitude et diminuerait graduellement lorsque l'altitude de ces terrains augmente.

## Introduction

Fasciolosis is a worldwide parasitosis caused by the liver fluke *Fasciola hepatica* Linnaeus, 1758 [30]. It affects humans, but its main definitive hosts are ruminants such as cattle and sheep [37–38]. In most countries of the world where these ruminants are reared, this disease is responsible for significant economic losses [25]. The transmission of the disease to other definitive hosts requires a freshwater gastropod, which ensures the development of *F. hepatica* larval forms until the emission of cercariae. The latter become attached to vegetation and turn into metacercariae, thus becoming infectious forms [5]. In Western Europe, *Galba truncatula* O.F. Müller, 1774 [41] is the common snail host of *F. hepatica* [14,57]. However, other species of Lymnaeidae such as *Omphiscola glabra* O.F. Müller, 1774 [41] are also capable, to varying degrees, of being intermediate hosts [7,38,65].

The development of *G. truncatula* populations and, consequently, of *F. hepatica* larval forms is dependent on the climate in the region or country where this lymnaeid lives. The most favourable conditions are temperatures ranging between 10 °C and 25 °C and high relative humidity depending on atmospheric precipitation. As a result, the disease is common in temperate regions like most European countries. This relationship between climate and the parasitosis was demonstrated by the observations of Weybridge researchers in England [44–46]. Other authors have broadened this relationship between parasitosis and climate by incorporating other factors such as vegetation growth, local climatic variations (microclimate) and pasture topography [34–35]. The existence of this relationship has since been verified by numerous authors [15,39,47].

The relationship between climate and fasciolosis has led to the development of predictive models to estimate the risk related to this disease. Using different approaches, including the Geographical Information System, several authors have specified endemic areas for fasciolosis in different countries of the world such as Brazil [3], Bolivia [17], Cambodia [62], Ethiopia [66], Iran [22], Ireland [60], Sweden [42], Switzerland [6], the United Kingdom [15] or the United States [34]. Several climatic forecast indices were also used to indicate the chances of disease transmission for each month of the year. Moreover, the accumulated values of these climate risk indices allowed researchers to define the beginning and end of fasciolosis transmission for each year and the classification of endemic zones by low, moderate and high degrees of risk [18]. Most models were mainly validated by means of surveys on the prevalence of *F. hepatica* infection in the definitive host [33,63]. In contrast, those using data on the intermediate host are much less numerous [6,48].

In France, no map delimiting areas at risk for fasciolosis has yet been published. According to Chauvin et al. [10], these risk areas are represented by all the water points where the host snail lives. However, the main outbreaks of the animal disease were in breeding areas such as Burgundy, Centre, Lorraine, Normandy, South-West and Vendee [9,19]. The Limousin region is also affected by the disease since cases of fasciolosis have been reported in the last twenty years. Cases of human fasciolosis were detected between 1955 and 1998 in 860 people, most of them living in Limousin [55–56]. A prevalence of 13% (out of 12,724 cattle examined between 1983 and 1985) was reported by Mage [31] in the same area. This value has been confirmed in another paper by Mage et al. [32], reporting a prevalence of 17.1% (out of 12,389 cattle) in the department of Corrèze between 1990 and 1999. As the climatic conditions prevailing in this region are favourable for the development of *G. truncatula* populations [14], it was interesting to determine whether there were variations in the risk intensity for fasciolosis via the following two questions: did the native populations of *G. truncatula* show variations in the prevalence of their natural infection with *F. hepatica* in relation to the relief and climatic conditions of the Limousin municipalities? Could areas at risk for fasciolosis be identified in this region based on the natural infection of snails? To answer these two questions, a retrospective study was carried out on snails that our team collected between 1970 and 2006 from pastures of farms with reported animal fasciolosis or from wild watercress beds at the origin of human fasciolosis cases. Other data were obtained from samples taken from farms in which snails have been experimentally transplanted. Among the three departments of Limousin, the Haute-Vienne was chosen for this study because of numerous samples of snails collected by our team in this area from the 1970s. The results obtained in several farms or in several wild watercress beds have already been reported [1–2,32,52]. In contrast, the data on the natural infection of other snail populations have not yet been published.

## Materials and methods

### Study area

**Figure 1:**
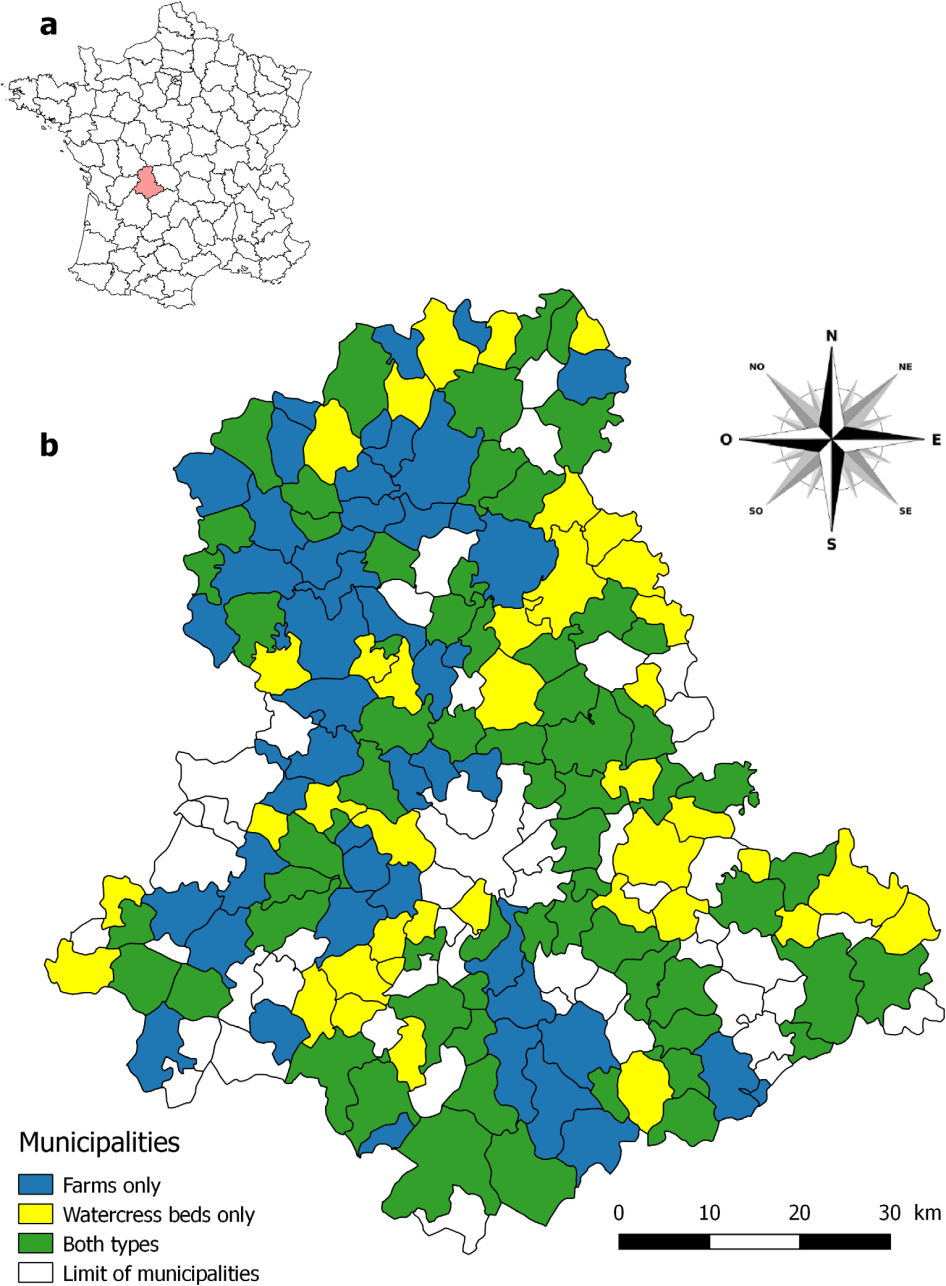
Geographical location of the department of Haute-Vienne in central France (a) and the 151 municipalities on which the 259 farms and 121 wild watercress beds are located (b). No snail samples were taken in municipalities in white.

The department is located in the north-western part of the Massif Central (Fig 1). The latitude of this area ranges from 45°26' to 46°21' N, while its longitude ranges from 0°48' to 1°34' E (https://www.coordonnees-gps.fr). Its surface area is about 5520 km^2^ and its altitude varies between 160 m in the valley of the Vienne and 777 m at the Crozat Mount, located near the lake of Vassivière. The department is not really divided into distinct geographical entities, but three large groups can be identified: the Basse Marche (altitude, 150-300 m) in the northern third, the valleys of Vienne and its tributaries (altitude, 150-400 m) largely located in the central part, and the plateaus and mounts of Limousin (altitude, 300-777 m) along the eastern, south-eastern and south-western borders of the department [11]. The Haute-Vienne is constituted of crystalline grounds dating back to the primary era and these grounds were folded during the formation of this Hercynian chain. The subsoil is mainly composed of granite or gneiss, with some outcrops of mica-schist or serpentinite. The result is the presence of numerous rivers: more than 7000 km, of which the main one is the Vienne. Owing to the nature of soils, the pH of running water ranges from 5.6 to 7 and the level of dissolved calcium is generally less than 20 mg/L [21]. The continental type climate is attenuated by moist winds coming from the Atlantic Ocean. However, in its eastern part, the climate undergoes a mountainous influence due to the proximity of the Massif Central [11]. The Haute-Vienne is predominantly rural and the human activity is mainly focused on livestock and silviculture: the department comprises 168,000 ha of natural grassland and 149,996 ha of wood, which corresponds to 33.1% and 29.6% of its area, respectively.

### Snail populations

The populations selected for this study are those in which regular or irregular sampling of *G. truncatula* occurred over several consecutive years. We did not take into account snail habitats in which occasional sampling was carried out to detect a natural infection of snails or to perform experimental infections in the laboratory. Table 1 shows the type of habitats colonized by *G. truncatula*, the dates of these investigations and the total number of snail samples collected. Figure 1 shows the 151 municipalities of the department on which these samples were taken. Two habitat groups were considered. The first group was represented by snail habitats present in the swampy grasslands of 259 farms breeding cattle or sheep. The first 234 were prospected for the first time between 1970 and 1999 when one or more cases of animal fasciolosis were detected by the local veterinarians. They were again investigated between 1976 and 2006 when new cases of fasciolosis were discovered. Each investigation was carried out in March or April because all snail habitats are then waterlogged [58] and a sample of snails was taken each time to look for larval forms of *F. hepatica*. The remaining 25 farms were selected because of a field experiment carried out by our team on transplantations of snails from their original habitats into new areas [64]. Snail sampling was also performed each year in March between 1986 and 1995. The second group of habitats concerns wild watercress beds at the origin of human fasciolosis cases [50,54–55]. Forty-five of these beds were first prospected between 1974 and 1989, following the discovery of one or more cases of human distomatosis and one or two samples of *G. truncatula* were taken from each bed in subsequent years to determine the prevalence of *F. hepatica* infection in snails. Each bed was then reinvestigated each year from 1990 to 2006 to collect samples of snails and follow up on their parasitic contamination [14,57]. The other 76 beds were irregularly followed up between 1974 and 1998, with four snail samples taken during this period to detect possible changes in the prevalence of their natural infection with *F. hepatica* [50–51,56]. In both cases, snails were collected in June or early July.

**Table 1:** Main characteristics of the different snail habitats, with indications of the number of snail samples.

Type of snail habitat (number of farms or beds)	Period of study	Snail samples		Number of snails per sample
			
		Number per farm or per bed	Total number	
Grasslands on farms				
Systematic survey for fasciolosis (234)	1970-2006	2, 3 or 4	515	From 100 to 150 (several habitats per farm)
Experimental transplantations (25)	1986-1995	10	250	50 or 100 (a single habitat per farm)*
Wild watercress beds				
Regular sampling (45)	1974-1989, 1990-2006	1 or 2, 15	68, 675	From 10 to 50 per bed*
Irregular sampling (76)	1974-1998	4	304	From 10 to 50 per bed*
Totals	–	–	1812	

* Depending of the size of the snail population.

### Snail investigations

Only adult lymnaeids (from 4-5 to 8-9 mm shell height) belonging to the overwintering generation were collected in these samples. The juveniles and pre-adults were not taken into account so as not to endanger the survival of each population. On each farm surveyed for fasciolosis, snail habitats were detected using the indicator plant method [58] and snails were randomly searched at sight in open drainage furrows and spring heads present in grasslands to obtain a final sample of 100-150 individuals per farm (with a number of snails ranging from 14-18 to 25-30 adults per habitat depending on its area). In each of the other 25 farms, snail habitats were found using the same method and a sample of 50 or 100 snails depending of the size of the population was taken from the same habitat (Table 1). The choice of March or April for sampling was dependent on the altitude of the habitats and the period during which cercarial shedding occurred in the Limousin region: April or May, respectively [14,57]. In the wild watercress beds, the number of snails taken in each sample (from 10 to 50 adults per habitat) was dependent on the size of the snail population (Table 1). It also depended on the altitude of these beds and the date of cercarial shedding in the natural environment (July or August, respectively).

After their collection, the snails were transported on wet grass to the laboratory under constant conditions (generally 20 °C). They were then dissected under a stereomicroscope to find larval forms of *F. hepatica* (rediae and/or cercariae). The rediae of *F. hepatica* have been differentiated from those of *Calicophoron daubneyi* over the length of their body, the presence of appendages (*F. hepatica*) and the morphology of their pharynx [14,57]. The cercariae of *F. hepatica* have also been identified on their presence in the body of their rediae when they are well differentiated (the procercariae of *C. daubneyi* emerge very early from the body of their parental rediae and finish their differentiation in the visceral cavity of the snail) and on their characteristic white colour [14,57]. Snails having only sporocyst(s) in their bodies were not counted because the sporocyst shape and its dimensions do not allow us to identify the digenean species in most cases. No molecular biology study was carried out during the period of snail investigations (1970-2006) to detect the presence of the parasite within the snail.

### Geographical and climatic data

The mean altitude of each municipality was obtained by considering that of its main city and was determined using the Carte-de-France website (http://www.cartesfrance.fr). The other two variables were the mean annual rainfall and the mean annual temperature from 1971 to 2000, and came from maps published by Météo France [40].

### Statistical analyses

**Figure 2:**
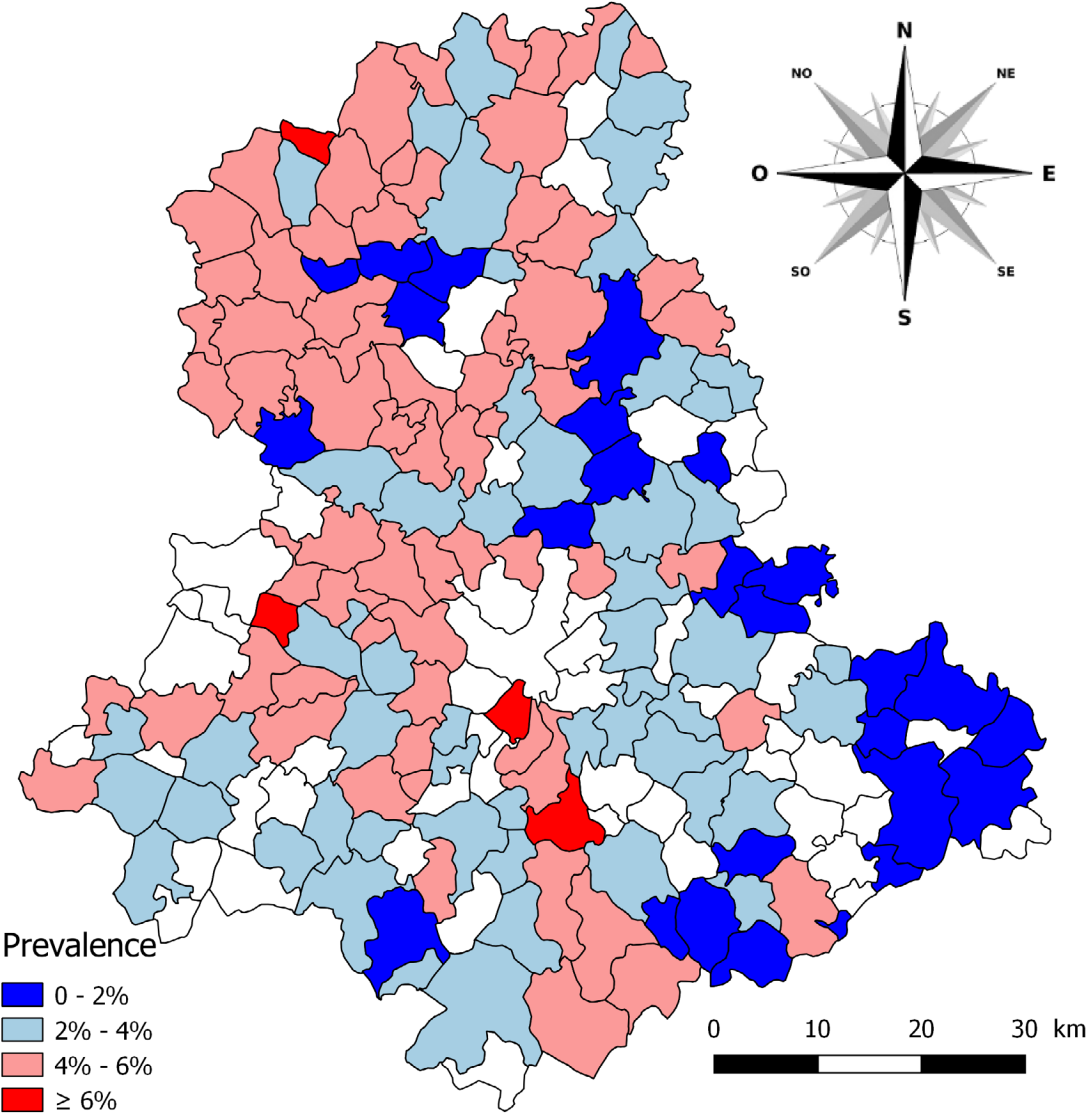
Prevalence of natural infection with *Fasciola hepatica* in the 151 municipalities of the Haute-Vienne department on which snail samples were collected between 1970 and 2006. No snail samples were taken in municipalities in white.

The prevalence of *F. hepatica* infection was calculated using the ratio between the number of infected snails and that of adult snails collected from a farm or a watercress bed. The individual values obtained for the different farms and watercress beds in a given municipality were then pooled to obtain a mean prevalence. The results are shown in Figure 2.

Different categories for the mean altitude of municipalities, their mean annual rainfall or their mean annual temperature were used according to the data provided by the Carte-de-France website (altitude) or the maps published by Météo France [40]. These categories have been chosen so that they have identical amplitude. As the altitude of farms and watercress beds ranged from 160 m to 570 m, the individual values noted for the prevalence of natural infection in snails were classified in the following four categories of altitude: < 300 m, 300-400 m, 401-500 m, and > 500 m. Five categories were also used for mean annual rainfall (<900 mm, 900-1000 mm, 1001-1100 mm, 1101-1200 mm, and > 1200 mm), whereas five other categories (<9.5 °C, 9.5-10 °C, 10.1-10.5 °C, 10.6-11 °C, and > 11 °C) were used for the mean annual temperature. Values for altitude, annual rainfall and annual temperature were first subjected to a Pearson's correlation test to assess the degree of relationship between these three parameters. As the latter were strongly correlated with each other (Table 3), the values given in the above categories of altitude, rainfall or temperature were subjected to a simple linear regression instead of being processed by multiple linear regression. The different analyses were performed using the R 3.3.0 software [49]. All prevalence values are given with their 95% confidence intervals.

**Table 3:** The results provided by the Pearson's correlation test when analysing values for the mean altitude, mean annual rainfall and mean annual temperature in the various municipalities of Haute-Vienne. CI, confidence interval.

Pearson's correlation coefficients (95% CI)			

Parameters	Mean altitude	Mean annual rainfall	Mean annual temperature
Mean altitude	1	0.794	-0.749
		(0.726;0.846) ***	(-0.812;-0.670) ***
Mean annual rainfall	0.794	1	-0.735
	(0.726;0.846) ***		(-0.800;-0.652) ***
Mean annual temperature	-0.749	-0.735	1
	(-0.812;-0.670) ***	(-0.800;-0.652) ***	

*** *p* < 0.001.

## Results

Out of a total of 108,481 snails collected in 151 municipalities, the overall prevalence of infection was 3.8% with insignificant differences between percentages found in snails from grasslands (3.1%-3.9%) and those from watercress beds (3.7%-4%, Table 2). In contrast, this prevalence varied according to municipalities with a minimum of 1% at Domps and a maximum of 7.4% at Saint-Jean-Ligoure. Figure 2 shows the distribution of these percentages in the 151 municipalities. Prevalences between 4% and 6% were found in most municipalities of Haute-Vienne, irrespective of their geographical location. Values above 6% were recorded in only four municipalities in the west and the south of this department. In contrast, the lowest values (<2%) were noted for the municipalities (in dark blue) located in the Blond Mounts (north-western part of Haute-Vienne), the Ambazac Mounts (north-eastern part) and the Limousin Mounts located along the eastern, south-eastern and south-western borders of the department. This decrease in prevalence values was significantly correlated (*p* < 0.001) with the increasing altitude of municipalities, as shown in Table 4(prevalence) and 5 (linear regression). This model could explain 50.7% of total variance in the prevalence of snail infection with *F. hepatica*.

**Table 2:** Prevalence of *Fasciola hepatica* infection in snail samples collected from breeding farms and wild watercress beds in Haute-Vienne. CI, confidence interval.

Type of snail habitat (number of farms or beds)	Total number of snails		Prevalence in % [95% CI]
		
	collected	infected	
Grasslands on farms			
Systematic survey for fasciolosis (234)	67,129	2666	3.97 [3.82;4.12]
Experimental transplantations (25)	14,000	434	3.10 [2.81;3.40]
Wild watercress beds			
Regular sampling (45)	15,394	623	4.05 [3.74;4.37]
Irregular sampling (76)	11,958	442	3.70 [3.36;4.05]
Totals	108,481	4165	3.84 [3.72;3.96]

**Table 4:** Prevalence of natural infection with *Fasciola hepatica* in *Galba truncatula* collected from the department of Haute-Vienne between 1970 and 2006 in relation to different categories for the mean altitude, mean annual rainfall and mean annual temperature. CI, confidence interval.

Parameter and categories	Number of infected snails	Number of snails collected	Prevalence (%) of natural infection [95% CI]
Mean altitude			
< 300 m	1933	42,216	4.58 [4.38;4.78]
[300;400 m[	1883	47,501	3.96 [3.79;4.14]
[400;500 m[	317	16,620	1.91 [1.70;2.13]
≥500 m	32	2144	1.49 [1.02;2.10]
Total	4165	108,481	3.84 [3.72;3.96]
Mean annual rainfall			
< 900 mm	363	7679	4.73 [4.26;5.23]
[900;1000 mm[	1671	36,605	4.33 [4.12;4.54]
[1000;1100 mm[	1474	39,570	3.73 [3.54;3.92]
[1100;1200 mm[	602	19,983	3.01 [2.77;3.26]
≥ 1200 mm	55	2644	2.08 [1.57;2.70]
Total	4165	108,481	3.84 [3.72;3.96]
Mean annual temperature			
< 9.5 °C	1	100	1.00 [0.03;5.45]
[9.5;10 °C[	100	5255	1.90 [1.55;2.31]
[10;10.5 °C[	119	4235	2.81 [2.33;3.35]
[10.5;11 °C[	1509	41,437	3.64 [3.46;3.83]
≥ 11 °C	2436	57,454	4.24 [4.07;4.41]
Total	4165	108,481	3.84 [3.72;3.96]

**Figure 3:**
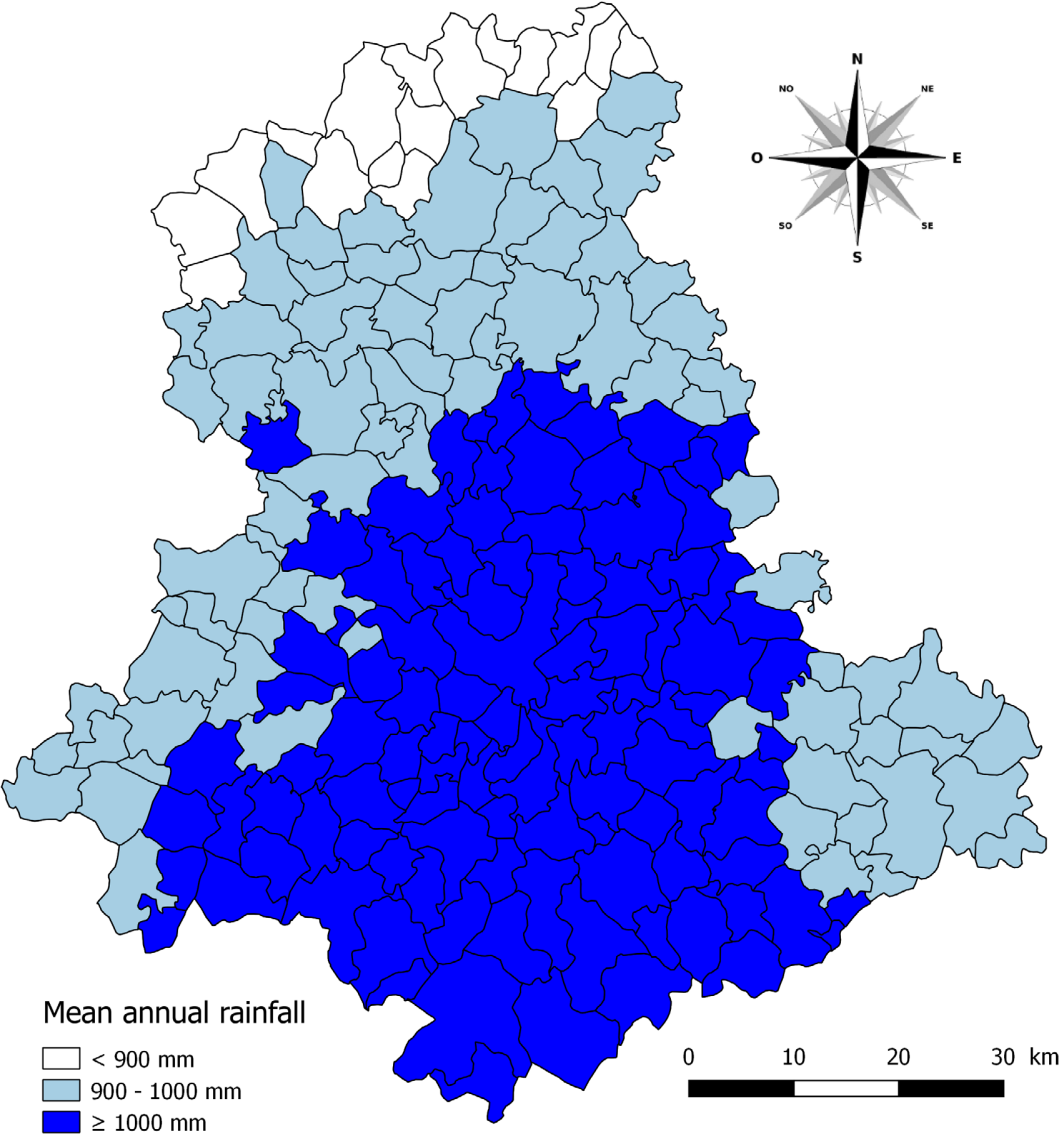
Mean annual rainfall from 1971 to 2000 in the 200 municipalities of the Haute-Vienne department.

**Figure 4:**
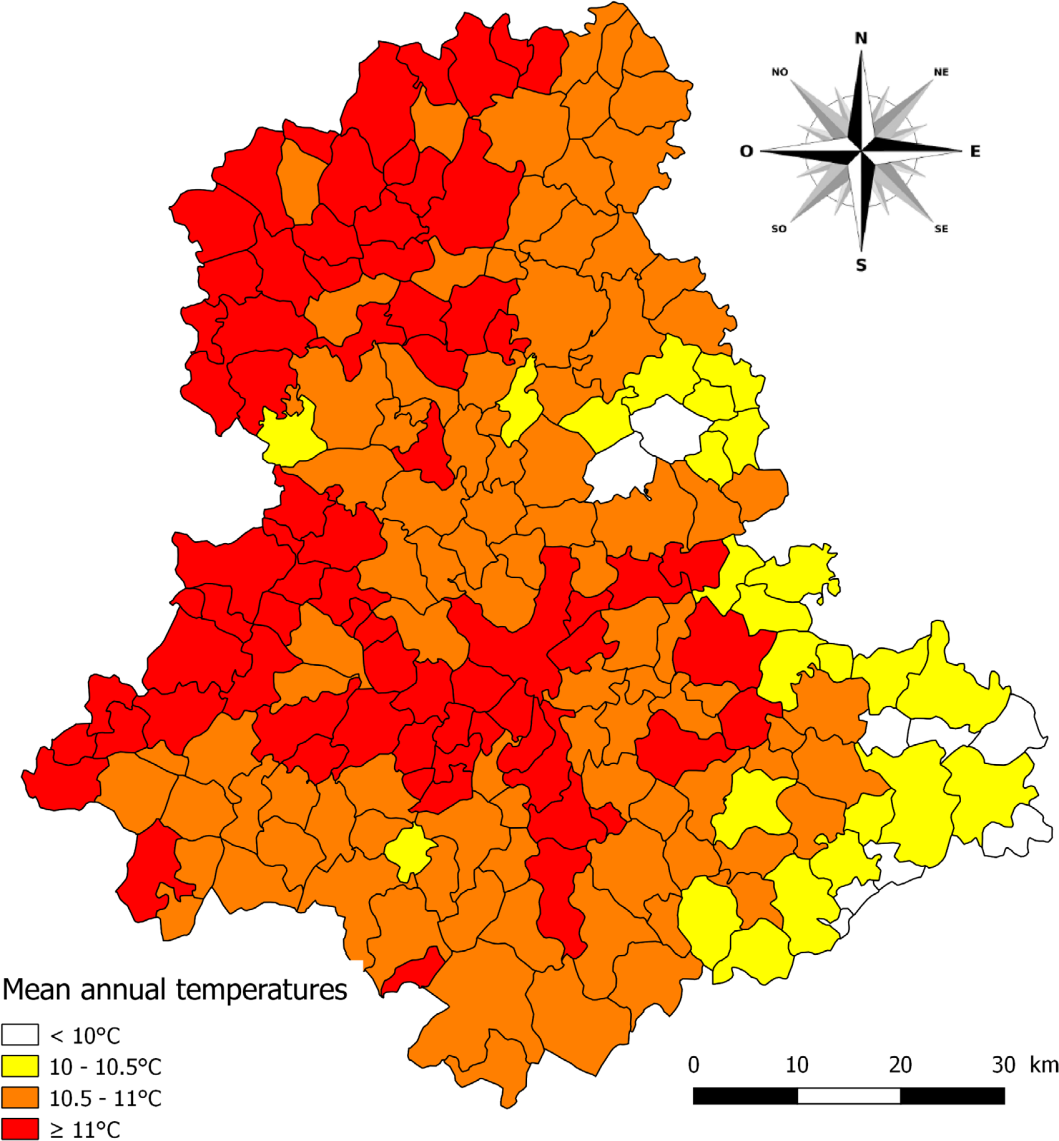
Mean annual temperature from 1971 to 2000 in the 200 municipalities of the Haute-Vienne department.

Figure 3 shows variations in mean annual rainfall in the 200 municipalities of Haute-Vienne. Annual rainfall was closely related to the mean altitude of the municipalities (Table 3). The lowest precipitations were noted in the municipalities at the northern limit of Haute-Vienne and these values increased in intensity towards the south of the department. The most abundant precipitations (>1200 mm per year) were noted in the communes on the eastern, south-eastern and south-western borders of the department, where the Limousin Mounts are located. A significant relationship (*p* < 0.001) between the decrease in prevalence in snails and the increase in mean annual precipitations was noted, as shown in Tables 4 and 5. However, this model based on the annual precipitations can only explain 25.2% of total variance in the prevalences recorded in *G. truncatula* (Table 5). On the other hand, the mean annual temperature (Figure 4) has an inverse distribution in the department, with the highest values in the municipalities located in the north and west, while the lower ones are on the eastern and south-eastern borders. There was also a significant (*p* < 0.001) and positive relationship between the increase in prevalence and that of the mean annual temperature in municipalities (Tables 4 and 5). The latter model explains 29.3% of total variance in the prevalences observed in snails (Table 5).

**Table 5:** Values provided by a simple linear regression when used to calculate the relationship between the prevalence of natural infection with *Fasciola hepatica* in snails and the mean altitude, mean annual rainfall or mean annual temperature in different municipalities of Haute-Vienne. Df, degrees of freedom.

Equation and coefficients	Estimate	Standard error (S.E.)	95% confidence intervals	*t* value	*p*-value
Prevalence = *a*.altitude + *b*					
* a*	-1.094 × 10^−4^	8.778 × 10^−6^	-1.268 × 10^−4^;-9.206 × 10^−5^	-12.46	*p* < 0.001
*b*	7.375 × 10^−2^	2.975 × 10^−3^	6.787 × 10^−2^;7.963 × 10^−2^	24.79	*p *< 0.001
Residual S.E.: 0.010; 149 df					
Multiple R^2^: 0.5104; adjusted R^2^: 0.5071					
Prevalence = *a*.rainfall + *b*					
* a*	-6.638 × 10^−5^	9.239 × 10^−6^	-8.463 × 10^−5^;-4.812 × 10^−5^	-7.184	*p* < 0.001
* b*	1.063 × 10^−1^	9.563 × 10^−3^	8.741 × 10^−2^;1.252 × 10^−1^	11.117	*p* < 0.001
Residual S.E.: 0.012; 149 df					
Multiple R^2^: 0.2573; adjusted R^2^: 0.2523					
Prevalence = *a*.temperature + *b*					
* a*	0.0125	0.002	0.009;0.016	7.964	*p* < 0.001
* b*	-0.098	0.017	-0.132;-0.065	-5.740	*p* < 0.001
Residual S.E.: 0.011; 149 df					
Multiple R^2^: 0.2986; adjusted R^2^: 0.2939					

When both types of breeding farms (cattle or sheep) are located in the same municipalities, no significant difference between prevalence values in snails was noted, regardless of the factor considered.

## Discussion

The overall prevalence of *F. hepatica* infection in adult snails collected in Haute-Vienne was 3.8% (Table 2) in the present study, with negligible differences between percentages observed in grasslands and those found in watercress beds. Different percentages were reported in Europe by the authors who dissected or analysed by molecular biology a large number of *G. truncatula* to find larval forms of *F. hepatica*. In Great Britain, the mean prevalence was less than 2% in more than 52,000 *G. truncatula* dissected by Ollerenshaw [43] between 1960 and 1969. In Spain, the prevalence was 11.4% in the 5486 snails dissected by Manga-González et al. [36], but was only 4.4% in the 1141 snails dissected by Iglesias-Piñeiro et al. [24]. A molecular biology study revealed a prevalence of 7.0% in 4733 *G. truncatula* collected from 70 Swiss farms [59]. In the French region of Limousin, the prevalence of natural infection in dissected snails was 5.1% in 18,791 *G. truncatula* from grasslands of 141 farms [32] and only 1.7% in 19,249 adult snails collected from 59 watercress beds [13,52]. The difference between our results and those of the above authors can be explained by the nature of the material studied (pre-adult snails, when collected, are usually more numerous than adults in a population of *G. truncatula*) and the method used to detect infected snails (the latter are more numerous in a molecular biology study due to the counting of snails containing only sporocysts of *F. hepatica*). However, another hypothesis, based on the existence of a higher parasitic pressure in the grasslands of farms which intensively bred their livestock since the 1970s [8,29], cannot be excluded to explain this prevalence of 3.8% reported in this study.

In Haute-Vienne, the prevalence of *F. hepatica* infection in snails significantly decreased with the increasing altitude of municipalities. The lowest prevalence values (<2%) were noted in municipalities whose altitude exceeds 400 m and more. This result is rather difficult to interpret due to a relative lack of information on this point in the literature. Rapsch et al. [48] reported that the risk of *F. hepatica* infection in snails and consequently in the definitive host progressively decreased with the altitude of snail habitats: at 2000 m, the risk is reduced by 50% and becomes negligible at 2600 m. The risk of fasciolosis in the definitive host also decreased with increasing altitude in some countries such as southern Brazil [4,16] or Uganda [23], and this would likely be due *i)* to a growing paucity of intermediate hosts when the altitude of areas increases, and/or *ii)* to lower temperatures at higher altitudes, allowing only slower development of intramolluscan stages. The difference between our results and those of the above authors is that the altitude classes used in this study range from 200 to 600 m, while those used by the authors are considerably larger (from 1139 m to 3997 m for Howell et al. [23], for example). Under these conditions, one may wonder what factors act on the natural infection of *G. truncatula* when the altitude of its habitats exceeds 400 m. The lower size of snail populations in these areas [53] and the hypothesis of a lower susceptibility of these *G. truncatula* to *F. hepatica* miracidia may partially explain this decrease in the prevalence of natural infection. The existence of specific ecological conditions for lymnaeids in municipalities between 400 and 600 m, due to the predominance of conifers and perhaps to a greater acidification of running water, can also be proposed to explain our results.

To complete the life cycle of the parasite, the environment must provide a consistent set of suitable conditions of temperature and moisture for the development of the larval stages and the development of the intermediate host itself [61]. In this study, the prevalence of natural *F. hepatica* infection in *G. truncatula* decreased with lower mean annual temperatures in the municipalities, or with the increase in their average annual rainfall. The effect of temperature on the development of *G. truncatula* has been known for some time because a minimum temperature of 10 °C is necessary for the growth of the snail [27]. Similarly, the larval forms of the parasite do not develop in snails below 10 °C [28]. The negative effect of this climatic factor on the prevalence of *F. hepatica* infection is rather difficult to interpret. Among the hypotheses that may explain this result, the most reliable is to attribute this to the delay that an increase in altitude and, consequently, a decrease in the mean annual temperature cause on the growth of vegetation and biology of micro-invertebrates. The period during which the miracidial infection of snails takes place would be reduced in spring and autumn because of winter conditions that are longer above 400 or 500 m in altitude. An argument supporting this hypothesis is the existence of a single annual generation (instead of two per year usually) for *G. truncatula* in the department of Creuse, near Haute-Vienne, above 500 m [12] or in the Jura and the Alps, when altitude rises [52]. However, the possibility of a lower *Fasciola* infection in cattle or sheep herds when altitude rises, as demonstrated in Brazil [16] or in Uganda [23], cannot be completely excluded. In contrast to temperature, an increase in annual precipitations (from < 900 to > 1200 mm per year) has a negative effect on the natural infection of snails with *F. hepatica* when they live on acid soils, even though this climatic factor is strongly correlated with the altitude of habitats and the temperature. This demonstrates that an excess in annual precipitations, as well as the drying up of the environment in which snails live [26], has a restrictive effect on the infection of snails by the parasite. Two hypotheses can be proposed to explain this last result. The first is to consider a greater dissemination of miracidia due to this excess of water and, consequently, a lower contamination of *G. truncatula* by *F. hepatica*. The second hypothesis is related to a greater acidification of these soils on granite or gneiss when a surplus of atmospheric precipitations occurs [20], which would not allow optimal development of *G. truncatula* populations but would affect their infection by modifying the performances of miracidia or by eliminating them.

In conclusion, the prevalence of *F. hepatica* infection in *G. truncatula* shows a significant decrease when the altitude of their habitats and the mean annual precipitations rise, or when the mean annual temperature decreases. Studying the prevalence of infection in these snails thus makes it possible to delineate zones at risk for fasciolosis on the acid soils of Haute-Vienne. The risk of infection for livestock would be greater in areas below 400 m in altitude and would gradually decrease when the altitude of the land increases. Further observations are needed to confirm these results in other French regions or other temperate countries on acid soils.

### Conflict of interest

The authors declare that they have no conflicts of interest in relation to this article.
